# Editorial: New Trends of Substance Abuse: Looking for New Psychotropic Effects of Chem Sex Drugs, Cognitive Enhancers, and New Psychoactive Substances

**DOI:** 10.3389/fpsyt.2020.612192

**Published:** 2020-11-23

**Authors:** Simona Pichini, Annagiulia Di Trana, Marta Torrens, Norbert Scherbaum, Simona Zaami

**Affiliations:** ^1^National Centre on Addiction and Doping, Istituto Superiore di Sanità, Rome, Italy; ^2^Unit of Forensic Toxicology, Section of Legal Medicine, Department of Excellence of Biomedical Sciences and Public Health, Polytechnic University of Marche, Ancona, Italy; ^3^Departament de Neuropsiquiatria, Institut Hospital del Mar d'Investigacions Mèdiques, Barcelona, Spain; ^4^Department of Psychiatry and Psychotherapy, Medical Faculty, LVR-Hospital Essen, University of Duisburg-Essen, Essen, Germany; ^5^Unit of Forensic Toxicology, Section of Legal Medicine, Department of Anatomical, Histological, Forensic, and Orthopedic Sciences, Sapienza University of Rome, Rome, Italy

**Keywords:** chem sex drugs, cognitive enhancers, psychoactive substances, psychotropic effects, new trend of substance abuse

In the last few years, a wide range of pharmacologically active substances entered the legal and illegal market of abused compounds creating new challenges for health professionals dealing with users and their health threats (1–6). Among the increasing number of abused substances, we can include not only new psychoactive substances (NPS), but also chemsex drugs, and cognitive enhancers (7–10).

Chemsex drugs are a group of psychoactive substances (e.g., cocaine, methamphetamine, synthetic cathinones, and synthetic cannabinoids and gamma hydroxybutyric acid, GHB, GBL) and non-psychoactive drugs (e.g., erectile dysfunction agents and alkyl nitrites) intentionally or non-intentionally used in the context of sexual intercourses, most commonly among gay and bisexual men (7, 8). The reported simultaneous use of more than one substance at a time has been reported to entail medical and psychiatric complications, such as psychosis, aggressive behavior, and suicidal ideation.

Similarly, cognitive enhancers are used in medical practice as medication to treat specific cognition deficits in mental diseases, such as attention deficit hyperactivity disorder and Alzheimer's disease. In contrast, their non-medical use has been spreading worldwide due to their alleged capability to increase mental alertness and concentration, as well as to boost memory and energy levels. Although the psychostimulant effects of these substances are necessary to treat cognitive impairment, the psychiatric/psychotropic effects of their misuse, and the consequences for mental health, are poorly described (9, 10).

Generally speaking, unlike what happened in the past century, every year a non-negligible number of new psychoactive substances appear in the illegal market. Their permanence both on the market and on web-based channels only depends on “consumers' satisfaction”: a balance between number and length of positive subjective effects vs. acute side effects (11). Whereas, both outcomes can be reported by the same users, e.g., in web forums, short- and long-term psychiatric effects cannot be specifically identified and described due to the speed at which such substances enter and leave traffic channels.

This Research Topic seeks to provide updated studies, reviews, mini reviews, opinion papers, perspective articles, and case reports on psychiatric and psychotropic effects of chemsex drugs, cognitive enhancers, and more generally on new psychoactive substances. This collection aims to shed light on this incoming hazard to scientists and health professionals operating in the field of psychiatric damages caused by drug abuse.

As above reported, evidence on the Chemsex drugs' influence on mental health were provided by Bohn et al.. The research group administered to volunteers from the German MSM community a survey concerning recreational substance use, substance use in a sexual setting, mental health, adverse consequences of Chemsex behavior, and others. They concluded that the consumers of methamphetamine, mephedrone, GHB and or ketamine in sexual settings presented several more symptoms of depression, anxiety and somatization than the non-chemsex abusers and German general population.

Besides, Pereira et al. investigated the effects of regular GHB consumption and GHB-induced comas on the human brain structure and impulsivity. The study showed that only multiple GHB-induced comas could affect the microstructural tissue in the white matter, that is alleged to be the cause of the higher self-reported impulsivity.

An emerging phenomenon associated to Chemsex is the so-called “slam,” being the practice of injecting drugs in Chemsex contests. A study on the cathinones injection in slam context was presented by Schreck et al. with the aim to evaluate the presence of the cathinone use disorder. Based on 39 “slam” notifications, Cathinone related disorder appeared particularly important in this population of users due to the negative consequences of slam practice.

The problem of stigmatization of drug addiction and the consequent social exclusion is discussed by Brown. After an historical overview of main drug related social issues, the author identified in the emerging phenomenon of Chemsex the chance to do better, preventing the dehumanization of the MSM communities which could feed the drug abuse among MSM communities.

The marked increase of the prescription of substances like methylphenidate has recently raised concerns, suggesting a possible misuse of these substances to obtain mind-booster effects and create new niches in the NPS market (8). In fact, Napoletano et al. outlined the online availability of cognitive enhancers, comparing the information on online fora and the EMCDDA and UNODC NPS databases. Through the website NPSfinder®, 142 cognitive enhancers not reported in the official databases were individuated.

Besides recreational consumption, cocaine is also abused for social or cognitive enhancement. The motivation pushing people to take cocaine to self-medicate deficits in cognitive abilities was assessed by Kexel et al. in a sample of 42 cocaine users, divided into a non-social motive group and a social motive group. Then, the social motive group was compared to a control group to assess their cognitive functioning through a comprehensive neuropsychological test. The study confirmed that people who use cocaine as a cognitive enhancer have considerable deficits in cognitive empathy and working memory. Currently, different treatments for cocaine addiction have been proposed in the literature. The long-term outcomes of an alternative approach were evaluated by Madeo et al. in their retrospective observational study. A large cohort of cocaine addicts who underwent repetitive transcranial magnetic stimulation (rTMS) for 3 months were observed and regular toxicological analysis of urine were conducted. As already observed in smaller samples, the rTMS shows promising results in the treatment of cocaine addiction.

The ethical implications of cognitive enhancers prescription were discussed by Zaami, Tagliabracci et al. and Ricci, who both focused their attention on the effectiveness of cosmetic neurology and the possible misuse to obtain “super-human” performances in healthy subjects. Although several opinions have been expressed by the international bioethical committee, the issue is still controversial and official guidelines are required by physicians. On the other hand, Schleim proposed a different approach to the cognitive enhancers issue, defining it as instrumental drug use. To this purpose, a brief discussion on the most relevant research and anecdotal evidence was reported by the author to corroborate his thesis.

The NPS issue is an everchanging reality, always posing new challenges to the scientific community and professionals working against this phenomenon. Investigating the NPS and other drugs use among patients in detoxification treatment in Germany, Specka et al. showed that polydrug consumption was common in that population, especially in opiate abusers, but NPS use in particular was infrequent and only short-term. The most commonly used NPS class in this population were synthetic cannabinoids. It is interesting to note that the most frequent reason for NPS use according to the participants, was that NPS were not included in the usual toxicological tests. Although this study shows that the NPS consumption is not so common. Giorgetti et al. drawn the attention toward the fact that a concerning number of NPS related deaths are reported in the literature. The research group focused on the post-mortem toxicology of synthetic cannabinoids-related fatalities. According to their research, the 26 cases reported are not sufficient to identify a typical toxic range for this class of substances and further analysis of post-mortem findings should be conducted to elucidate the toxicological profile of synthetic cannabinoids.

Corazza et al. conducted a phenomenological qualitative study to investigate the experiences of prisoners and professionals on “spice” consumption in prison, with the aims to elucidate the impact of NPS abuse on workers and people in custodial settings. Due to the frequent violent events experienced, the professionals involved in this study suggested to improve detection and treatment and support services in prisons together with enhanced training and education to face the problem.

Also, the psychiatric disorders caused by NPS abuse were investigated by Martinotti et al., who administered a questionnaire to a sample of inpatients hospitalized in Psychiatric Ward in Ibiza confirmed to have recently abused substances by toxicological analysis. According to their research, stimulants and psychodyspleptics drugs are the most popular club drugs and the polydrug consumption is a common practice of party goers. It is interesting to notice that the largest part of the considered population has already had a Bipolar Disorder diagnosis.

Nagy et al. assessed intake patterns of the cathinone alpha-pyrrolidinopropriophenone (a-PPP) in condition of prolonged free access to the drug, in an animal model. The authors concluded that a-PPP shows a limited abuse potential and/or support only recreational use patterns in rats.

On the other hand, NPS are also studied as safer alternatives to current therapies, such as in the case of mytraginine studied by Hassan et al. to treat morphine withdrawal symptoms in rats. After inducing morphine abstinence in the animals, the rats that showed withdrawal syndrome were treated with buprenorphine, methadone, or mytraginine and the effects were monitored and measured for 28 days. Although a high intraperitoneal dose of mytraginine was required (3–50 mg/kg), the substance showed a good potential as opiate withdrawal treatment compared to medication usually used in detoxification treatment.

Since the beginning of 2020, the COVID-19 pandemic has been changing the habits of the global population at every level. Zaami, Marinelli et al. analyzed the possible consequences of this unprecedent situation on the abusers' health, concluding by drawing the attention of the scientific community toward the importance of the psychiatric and psychological assistance to this fragile part of population.

In conclusion, this Research Topic is providing updated studies and reviews concerning the psychiatric and psychological implications of Chemsex drugs, cognitive enhancers and NPS abuse, highlighting the importance to continuously investigate this still unclear field by the scientific community and health professionals.

## Author Contributions

All authors listed have made a substantial, direct and intellectual contribution to the work, and approved it for publication.

## Conflict of Interest

The authors declare that the research was conducted in the absence of any commercial or financial relationships that could be construed as a potential conflict of interest.
